# Machine Learning Assisted Electronic/Ionic Skin Recognition of Thermal Stimuli and Mechanical Deformation for Soft Robots

**DOI:** 10.1002/advs.202401123

**Published:** 2024-06-12

**Authors:** Xuewei Shi, Alamusi Lee, Bo Yang, Huiming Ning, Haowen Liu, Kexu An, Hansheng Liao, Kaiyan Huang, Jie Wen, Xiaolin Luo, Lidan Zhang, Bin Gu, Ning Hu

**Affiliations:** ^1^ School of Mechanical Engineering Hebei University of Technology Tianjin 300401 China; ^2^ College of Aerospace Engineering Chongqing University Chongqing 400044 China; ^3^ School of Manufacturing Science and Engineering Southwest University of Science and Technology 59 Qinglong Road Mianyang 621010 China; ^4^ National Clinical Research Center for Chinese Medicine Acupuncture and Moxibustion First Teaching Hospital of Tianjin University of Traditional Chinese Medicine Tianjin 300381 China; ^5^ School of Basic Medicine Chongqing Medical University Chongqing 400042 China; ^6^ State Key Laboratory of Reliability and Intelligence Electrical Equipment Hebei University of Technology Tianjin 300130 China; ^7^ Key Laboratory of Advanced Intelligent Protective Equipment Technology Ministry of Education Hebei University of Technology Tianjin 300401 China

**Keywords:** electronic/ionic conductive hydrogel, machine learning, mechanical deformation, thermal stimuli

## Abstract

Soft robots have the advantage of adaptability and flexibility in various scenarios and tasks due to their inherent flexibility and mouldability, which makes them highly promising for real‐world applications. The development of electronic skin (E‐skin) perception systems is crucial for the advancement of soft robots. However, achieving both exteroceptive and proprioceptive capabilities in E‐skins, particularly in terms of decoupling and classifying sensing signals, remains a challenge. This study presents an E‐skin with mixed electronic and ionic conductivity that can simultaneously achieve exteroceptive and proprioceptive, based on the resistance response of conductive hydrogels. It is integrated with soft robots to enable state perception, with the sensed signals further decoded using the machine learning model of decision trees and random forest algorithms. The results demonstrate that the newly developed hydrogel sensing system can accurately predict attitude changes in soft robots when subjected to varying degrees of pressing, hot pressing, bending, twisting, and stretching. These findings that multifunctional hydrogels combine with machine learning to decode signals may serve as a basis for improving the sensing capabilities of intelligent soft robots in future advancements.

## Graphical abstract

A novel electronic skin (E‐skin) with mixed electronic and ionic conductivity can simultaneously achieve exteroceptive and proprioceptive. It is integrated with soft robots to enable state perception, with the sensed signals further decoded using the machine learning model of decision trees and random forest algorithms. This well‐designed hydrogel sensing system holds great potential for real‐world applications.

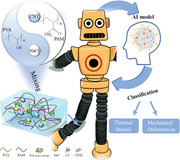

## 1 Introduction

Inspired by natural biological systems, soft robots have attracted significant attention from researchers due to their inherent softness and deformability.^[^ ^1^, ^2^ ^]^ More and more soft robots have been developed and have shown great potential for real‐world applications. Soft robots, like living creatures, require substantial perceptual capabilities for human‐robot interaction and high‐precision control.^[^ ^3^, ^4^, ^5^ ^]^ Electronic skin (E‐skin), as a critical component of sensing electronic devices, offers a promising solution for enhancing the perceptual capabilities of soft robots.^[^ ^6^, ^7^, ^8^ ^]^ However, current advancements in E‐skin mainly focus on perceiving external stimuli or proprioception, with only a few studies aiming to achieve both exteroception and proprioception. Realizing these capabilities imposes new requirements on the multifunctionality of E‐skin, including excellent mechanical properties,^[^ ^9^, ^10^ ^]^ high sensitivity,^[^ ^11^, ^12^, ^13^, ^14^ ^]^ wide sensing range,^[^ ^15^, ^16^, ^17^ ^]^ anti‐freezing,^[^ ^18^, ^19^, ^20^ ^]^ and thermal stimulus perception.^[^ ^21^, ^22^, ^23^ ^]^ Additionally, decoupling the abundant data obtained from the E‐skin and classifying the state of soft robots pose new challenges.^[^ ^24^ ^]^ Therefore, there is a strong demand for multifunctional E‐skin with exceptional signal processing capabilities to diversify the capabilities of soft robots.

Conductive hydrogels are well‐established flexible materials for perception in soft robots^[^ ^25^, ^26^ ^]^ and the prerequisite for their use in sensing applications is excellent stretchability.^[^ ^27^, ^28^, ^29^ ^]^ Conductive hydrogels achieve electronic conductivity and ionic conductivity by incorporating different conductive materials and thus utilize their piezoresistive properties to enable proprioception in soft robots.^[^ ^30^, ^31^ ^]^ Although electronic conductive hydrogels exhibit high sensitivity, their working range is typically limited to less than 200% strain as the conductive network breaks down with increased stretching. Conversely, ionic conductive hydrogels can endure significant tensile deformations but generally have lower gauge factors (GF) than 1. Soft robots require conductive hydrogels to have not only high sensitivity but also a wide sensing range. Thermal stimulus perception is equally important for hydrogel sensors in soft robot applications as it enables them to sense external stimuli.^[^ ^32^ ^]^ Due to the complex application scenarios of soft robots, the performance of hydrogels at low temperatures is also crucial for their sensing capability. For instance, water in the hydrogel tends to freeze at low temperatures, significantly affecting strain sensitivity, mechanical properties, and the service life of the hydrogel and thereby restricts its applications in harsh environments.^[^ ^33^ ^]^ To date, developing hydrogel sensors that possess all the critical features mentioned above remains a challenging task.

The advent of multifunctional hydrogel sensors has led to an increase in the volume and complexity of signal data, presenting new challenges in signal processing. Machine learning (ML) techniques have demonstrated exceptional proficiency in data classification and are well‐suited for sensing signal processing, particularly in accurately distinguishing small signal differences. The confluence of hydrogel sensors and ML has been extensively studied to enable advanced soft robots to intelligently perceive and perform tasks. For instance, Shu et al.^[^ ^34^ ^]^ utilized E‐skin and ML to identify various terrains, enhancing the exteroception capabilities of soft robots, while Yang et al.^[^ ^35^ ^]^ implemented an artificial neural network algorithm of long short‐term memory for motion detection of E‐skin. Therefore, the combination of ML techniques with hydrogel sensors holds great potential for enabling deformation sensing and thermal stimulus perception in soft robots, ultimately leading to the development of more intelligent E‐skin systems.

In this study, an innovative hydrogel (MNP) was synthesized by incorporating additives of multiwalled carbon nanotubes (MWCNT), sodium chloride (NaCl) and polyvinyl alcohol/poly‐acrylamide (PVA/PAM). Through manipulating the mass ratio of PVA and PAM, we were able to adjust the mechanical properties of the MNP hydrogel, making it suitable for use in diverse soft robots with varying elastic moduli. The addition of MWCNT and NaCl not only conferred electronic and ionic conductivity to the hydrogel, but also endowed the hydrogel with thermal stimulus sensing and anti‐freezing capabilities. Furthermore, a ML model based on decision trees and random forest algorithms was integrated for data analysis, enabling autonomous recognition of deformations and response to thermal stimuli. Experimental assessment was then performed to evaluate the mechanical properties, gauge factor, sensing range, anti‐freezing, and thermal stimulus response of the hydrogel. This work presents and validates a novel strategy for developing multifunctional hydrogel E‐skins characterized by their adjustable mechanical properties, hybrid conductive networks, advanced artificial intelligence, low cost, and high safety.

## 2 Results and Discussion

### 2.1 Synthetic Mechanism and Characterization of the MNP Hydrogel

**Figure 1:**
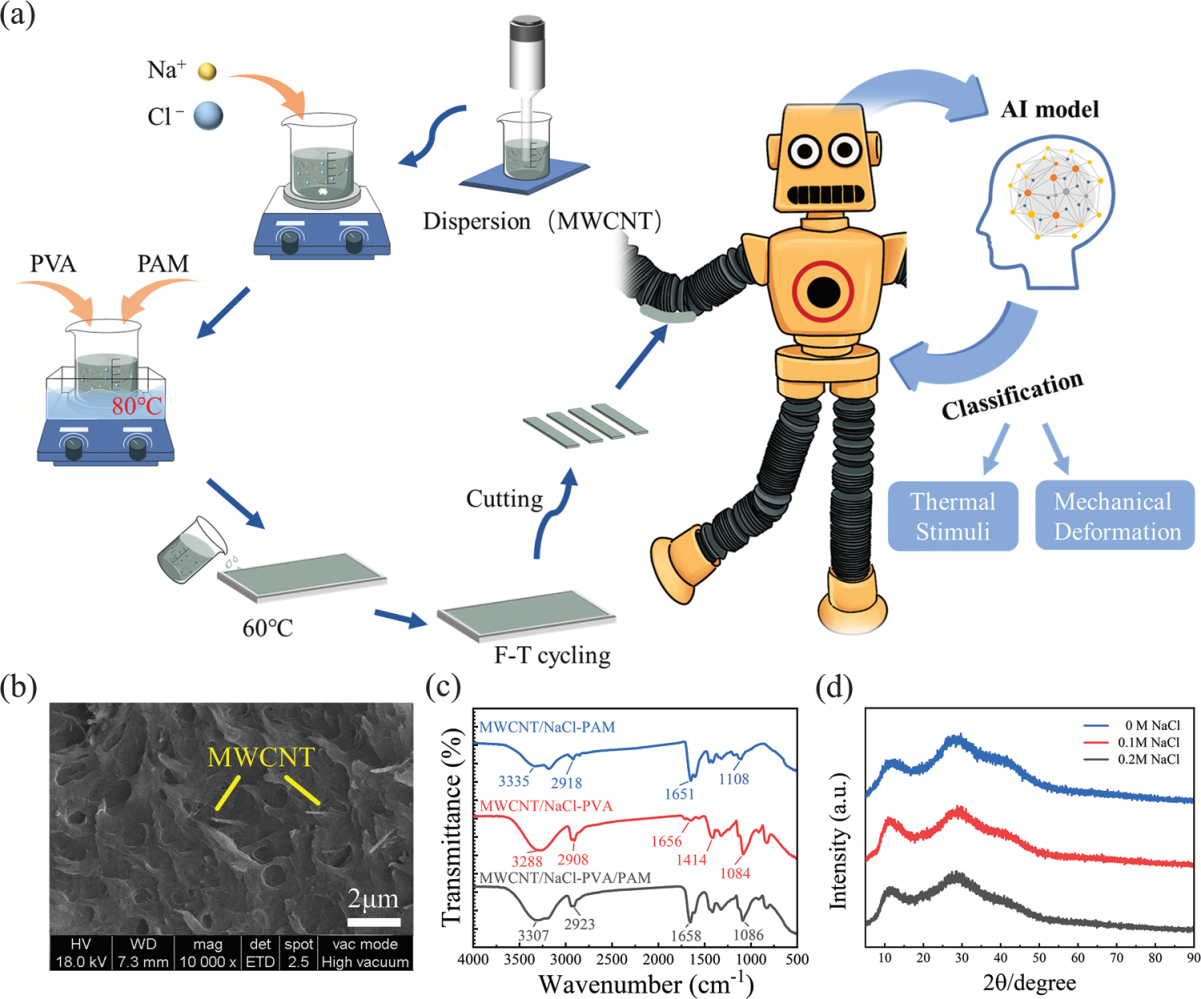
Synthesis and characterization of MNP hydrogels. a) Schematic diagram of the synthetic route of MNP hydrogels. b) SEM images of the MNP hydrogels. c) FTIR spectra of MWCNT/NaCl‐PAM, MWCNT/NaCl‐PVA and MWCNT/NaCl‐PVA/PAM hydrogels. d) XRD spectra of DN hydrogels with different NaCl contents (0, 0.1, and 0.2 m).

The preparation of the MNP double network (DN) hydrogel is schematically illustrated in **Figure** **1**a. MWCNT was homogeneously mixed with NaCl and added to the precursor solution of PVA and PAM to obtain the MNP hydrogel through chemical and physical cross‐linking. It was anticipated that PVA/PAM would form an interpenetrating network, with hydrogen bonds between inter‐ and intra‐molecular chains. MWCNT (Figures S1 and S2, Supporting Information) and NaCl served as conductive fillers in the hydrogels, providing both electron‐conducting paths and ion‐conducting paths. Figure 1b shows the microscopic morphology of the MNP hydrogels. The results indicated that the fracture surface of the PVA/PAM DN hydrogel displayed a heterogeneously interconnected mesh structure. The interpenetrating cross‐linking between PVA and PAM led to the formation of a 3D porous, dense, and uneven microstructure, with pore sizes ranging from 0.5 to 2 µm, allowing small Na^+^ and Cl^−^ ions to move directionally within these pores. Furthermore, the microscopic morphology revealed that the MWCNT was uniformly embedded within the 3D network of the hydrogel, forming an electron‐conducting pathway. To ensure homogeneity and prevent agglomeration, ultrasonication was employed during the MWCNT embedding process, which enhanced the sensitivity of sensing. The scanning electron microscope (SEM) measurements verified the interpenetrating cross‐linking between PVA and PAM and highlighted the microscopic 3D structure of the hydrogel, providing a conductive pathway for ions and electrons.

Fourier transform infrared spectrometry (FTIR) spectroscopy measurements are shown in Figure 1c. The FTIR spectrum of MWCNT/NaCl‐PAM exhibited bands at 3335 and 2918 cm^−1^, corresponding to the stretching vibrations of N─H and C─H groups, respectively. The band at 1651 cm^−1^ corresponds to the stretching vibration of C═O groups, while the band at 1108 cm^−1^ (C─C stretching) was also detected. The FTIR spectrum of MWCNT/NaCl‐PVA showed characteristic peaks at 3288 cm^−1^ (─OH) and 2908 cm^−1^ (─CH_2_). Additionally, bands were detected at 1656 cm^−1^ (C═O stretching), 1414 cm^−1^ (‐CH stretching), and 1084 cm^−1^ (C─O─O). The FTIR spectrum of MWCNT/NaCl‐PVA/PAM exhibited all the characteristic peaks of MWCNT/NaCl‐PAM and MWCNT/NaCl‐PVA. The spectra of the blends clearly show a noticeable increase in band intensity ≈3307 cm^−1^, indicating strong hydrogen bonding between PAM and PVA. The DN hydrogel was synthesized via in situ free radical polymerization in the water using homogeneous solutions of PVA and acrylamide (AM). It is highly likely that hydrogen bonding occurred between the ─OH groups of PVA chains and the ─CONH_2_ groups of PAM. In the FTIR spectrum of the DN hydrogels, all characteristic peaks of PVA and PAM are observed, indicating that the DN hydrogel has been successfully synthesized.

To further investigate the changes in crystallinity in the hydrogels, X‐ray diffractometer (XRD) measurements were performed (Figure 1d). The blunt diffraction peak of the MNP hydrogel appears at 2θ = 28.66°. The peak area decreases with increasing NaCl dosage, indicating suppression of hydrogel molecular chain crystallization due to chain‐entanglement. Meanwhile, previous studies have indicated that Na^+^ and Cl^−^ ions tend to become immobilized on polymer chains through dipole interactions, resulting in the formation of positively and negatively charged chain segments. The entanglement of such chain segments creates physical crosslinking, thereby improving mechanical properties.^[^ ^36^ ^]^ However, an excessive addition of NaCl can lead to the formation of white spots in the precursor solution, indicating limited compatibility between the PVA/PAM hydrogel and NaCl. Therefore, to strike a balance between mechanical performance and electrical conductivity of the MNP hydrogel, both of which are crucial for its overall performance, we maintained the NaCl concentration at 0.2 m in the subsequent study.

### 2.2 Mechanical Properties of the MNP Hydrogel

**Figure 2:**
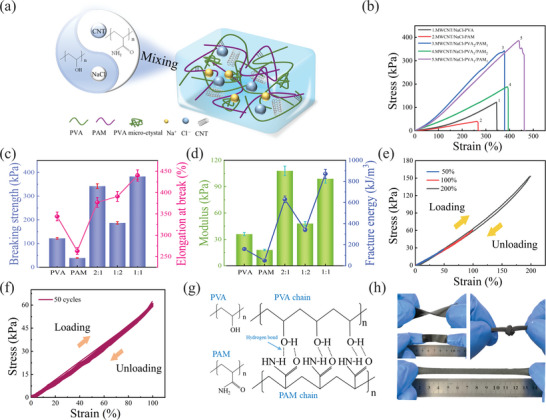
Tensile mechanical properties of MNP hydrogels. a) Microstructure schematic illustration. b) Tensile stress–strain curves of MNP hydrogels at different PVA to PAM mass ratios. c) Comparison of elongation at break and breaking strength of different hydrogels. Data are presented as mean ± SD (n = 3). d) Comparison of modulus and fracture energy of different hydrogels. Data are presented as mean ± SD (n = 3). e) Stress‐strain curves of MNP hydrogel in different tensile states (50%, 100%, and 200%). f) 50 stretching‐releasing mechanical cycle durability tests with 100% tensile strain. g) Schematic representation of PVA and PAM interspersed with cross‐linking. h) Visual representation of the physical structure of MNP hydrogels.

**Figure** **2** depicts the remarkable improvement in mechanical properties of the DN hydrogels compared to the single network (SN) hydrogels. Figure 2a schematically illustrates the microstructure of the MNP hydrogel, in which the chain entanglement between PVA and PAM leads to the formation of a denser molecular network. However, the mechanical properties of the DN hydrogels vary with the mass ratio of PVA and PAM. Various DN hydrogels were prepared with different mass ratios, namely 1:1, 1:2, and 2:1, denoted by PVA_1_/PAM_1_, PVA_1_/PAM_2_, PVA_2_/PAM_1_, respectively. For comparison, PVA and PAM SN hydrogels were also prepared. The typical tensile stress‐strain curves of these five hydrogels are presented in Figure 2b, while the corresponding tensile measurements are shown in Figures 2c,d. The elongation at break for PVA and PAM SN hydrogels were 344% and 263%, with breaking strengths of 122 and 39 kPa, respectively. Notably, the mechanical properties of the DN hydrogels exhibited significant improvement. Particularly, at a mass ratio of PVA to PAM of 1:1, the DN hydrogels demonstrated optimal mechanical properties, with elongation at break and breaking strength of 440% and 383 kPa, respectively. In comparison to PAM hydrogels, the modulus of PVA_1_/PAM_1_ hydrogels increased by 5.5 times from 18 to 99 kPa, while the fracture energy increased by 17.7 times from 49 to 871 kJ m^−3^. These tensile measurements indicate that the DN hydrogels exhibit superior mechanical properties at a mass ratio of PVA to PAM of 1:1.

The mechanical strength of the DN hydrogels is primarily governed by the chemical cross‐linking of AM, the self‐cross‐linking of PVA, and the interpenetration of PAM and PVA networks. Since the mechanical strength of PAM is relatively weak, a lower PVA content in the DN hydrogel hinders the effective formation of a crystallization region for the molecular chains of PVA, thereby resulting in poor mechanical properties. By increasing the overall PVA content, the elongation at break and breaking strength significantly improves. The presence of numerous hydroxyl groups in the PVA molecular chain facilitates cross‐linking through hydrogen bonding, thereby resulting in robust physical cross‐linking effects. Moreover, the increased PVA content promotes a denser arrangement of molecular chains, reducing the chain distance and promoting closer packing within the hydrogel. However, excessively high PVA content, while enhancing the modulus and breaking strength, reduces elongation break. Consequently, a mass ratio of PVA to PAM of 1:1 was adopted in subsequent tests.

Figure 2e demonstrates the stretching‐releasing curves of the MNP hydrogel under stepped cyclic stretched strain. Remarkably, the hydrogel exhibited almost complete recovery to its initial state at different strains (50%, 100%, and 200%) without displaying significant stress‐strain hysteresis loops, indicating excellent stress‐strain reversibility. Furthermore, Figure 2f illustrates that cyclic loading and unloading up to 100% strain for 50 cycles result in stress‐strain curves of loading and unloading exhibiting similar trends without significant hysteresis loops, affirming the mechanical stability and repeatability of the MNP hydrogel. The tensile cycling stability of hydrogels strongly depends on their microstructure. The FTIR spectra of the blends demonstrated strong hydrogen bonding between PAM and PVA. Hydrogen bonds form when hydrogen atoms within a molecule are attracted to more electronegative atoms (e.g., fluorine, oxygen, nitrogen, etc.). Therefore, the ─OH groups of PVA chains and the ɐCONH_2_ groups of the PAM chains form hydrogen bond links (Figure 2g). This interaction strengthens intermolecular bonding, resulting in a more tightly aligned molecular structure and improved mechanical stability of the material. These outstanding mechanical properties provide a solid foundation for the sensing application of the MNP hydrogels. The physical depiction presented in Figure 2h showcases the exceptional flexibility of the DN hydrogel synthesized in this study, enabling it to withstand significant deformations such as bending, twisting, and stretching.

### 2.3 Electrical Properties of the MNP Hydrogel

**Figure 3:**
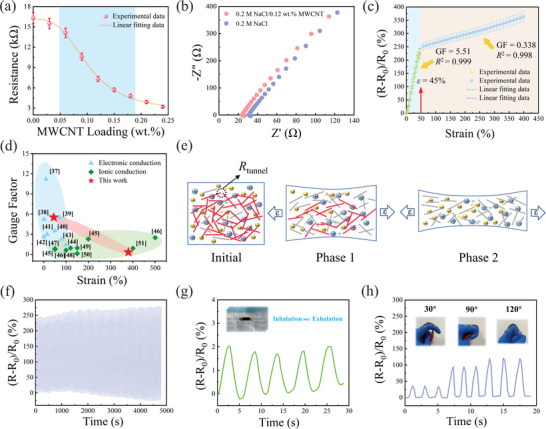
Electrical properties of MNP hydrogels. a) Hydrogel resistance at different MWCNT contents. Data are presented as mean ± SD (n = 3). b) Electrochemical impedance spectra of various hydrogels. c) Relative resistance changes rate‐strain curve. Data are presented as mean ± SD (n = 3). d) A scatter diagram showing the GF within some typical reported strain sensors.^[^ ^37^, ^38^, ^39^, ^40^, ^41^, ^42^, ^43^, ^44^, ^45^, ^46^, ^47^, ^48^, ^49^, ^50^, ^51^ ^]^ e) Schematic diagram of strain sensing. f) The loading‐unloading curves of 100 cycles (strain = 100%). g) Breathing monitoring. h) Recognition of finger bending.

Typically, the water within a hydrogel serves as a medium for ion transport, allowing for the movement of soluble salt ions. However, traditional methods of constructing hydrogels with ionic conductors have low gauge factors and are inadequate for small strain measurements. Through our previous research on carbon nanotube (CNT)/polymer composite strain sensors, we have observed that higher tunneling resistance within the conductive network, or a higher proportion of tunneling resistance to the total sensor resistance, leads to increased strain sensitivity. Consequently, doping the ionic hydrogel with CNT can enhance the sensitivity of the hydrogel to small strains. In our experiments, we added various amounts of MWCNT (0, 0.03, 0.06,…, 0.24 wt%) to the hydrogel with a NaCl content of 0.2. The addition of MWCNT resulted in a significant decrease in the resistance of the MNP hydrogel, as shown in **Figure** **3**a. Specifically, in the absence of MWCNT (0 wt%), the MNP hydrogel exhibited a resistance of 16.2 kΩ, which was attributed solely to the NaCl electrolyte conductivity. As the MWCNT content exceeded 0.05 wt%, there was a noticeable decrease in resistance with increasing MWCNT content in the hydrogels. The rate of resistance decrease slowed down when the MWCNT addition surpassed 0.18 wt%. These findings indicate that the percolation threshold of MWCNT in this study was ≈0.12 wt%. Furthermore, Figure 3b demonstrates that the ionic conductivity of the MNP hydrogel containing only NaCl was 2.57 S m^−1^, while the addition of MWCNT to the NaCl mixture increased the ionic conductivity to 3.55 S m^−1^. This suggests that MWCNT positively affected the ionic conductivity of the hydrogel. This may be due to the fact that the interface between the electronic and ionic conductors provides an additional migration path for ions, facilitating their transport through the mixed system and thus enhancing ionic conductivity. In other words, the strain sensing capability of the MNP hydrogel relies on the coupled conductivity of electrons and ions.

When the MNP hydrogel was stretched from 0 to 400%, it exhibited a substantial change in resistance, showcasing its ability to convert mechanical deformation into detectable resistance signals and demonstrating excellent sensing ability. The resistance changes during tensioning can be divided into two distinct phases: the first phase (0–45% strain) exhibited rapid changes in resistance, while the second phase (45–400% strain) demonstrated a slower rate of resistance change. As depicted in Figure 3c, the relative resistance change rate increased linearly with the increase in tensile strain in both phases (linearity *R* ^2^ = 0.999, *R* ^2^ = 0.998). In the strain range of 0–45%, the GF was determined to be 5.51, while in the strain range of 45–400%, the GF was 0.338. More importantly, compared to electronic conductors and ionic conductors, the MNP hydrogel showcased superior performance in terms of GF and strain range, surpassing most reported data in the literature (Figure 3d; Tables S1 and S2, Supporting Information). These excellent performances are likely attributed to the combined participation of electrons and ions in the conductivity mechanism. The detection of small mechanical deformations relies on electronic conductors, while the transport of soluble salt ions enables a wide strain sensing range, as the mechanical deformation intensifies, the electronic‐conducting network disconnects. By incorporating these two sensing mechanisms, high sensitivity can be achieved across a wide range. In the first phase, the involvement of MWCNT in the conductivity, along with the breakage of tunnel resistance, led to rapid changes in resistance. In the second phase, as the MWCNT conductive network disconnected due to the large deformation of the hydrogel, the changes in the ion water channels resulted in a decrease in ion concentration per unit volume and a change of the conductive path length, consequently increasing the resistance (Figure 3e). Additionally, Figure S3 (Supporting Information) displays the resistance change‐strain curve of the MNP hydrogel under stepped cyclic stretched strain at different strains (10%, 50%, 100%, and 200%). The curve revealed an almost complete recovery to the initial value, demonstrating the excellent reversibility of the strain‐sensing capability. Moreover, as shown in Figure 3f, when subjected to 100 cycles of loading and unloading strain (100%), the relative resistance change rate exhibited a consistent trend.

To investigate its ability to detect micro‐strains, we integrated the MNP hydrogel into a mask to monitor human breathing. Inhalation and exhalation caused micro‐strains within the hydrogel, which could be converted into detectable electrical signals. As depicted in Figure 3g, notable alterations in the resistance signal were observed while recording the airflow generated by the breathing of the testee over a 30 s duration. The measured respiration frequency correlated well with the actual values. Furthermore, in addition to monitoring micro‐strains, the MNP hydrogels were employed to monitor large strains, such as finger joint flexion. As shown in Figure 3h, the hydrogel sensor successfully recognized the bending angles of finger joints at 30°, 90°, and 120°. These practical applications highlight the remarkable potential of the hydrogel sensor in the field of deformation perception.

### 2.4 Anti‐Freezing and Thermosensitive Performance

**Figure 4:**
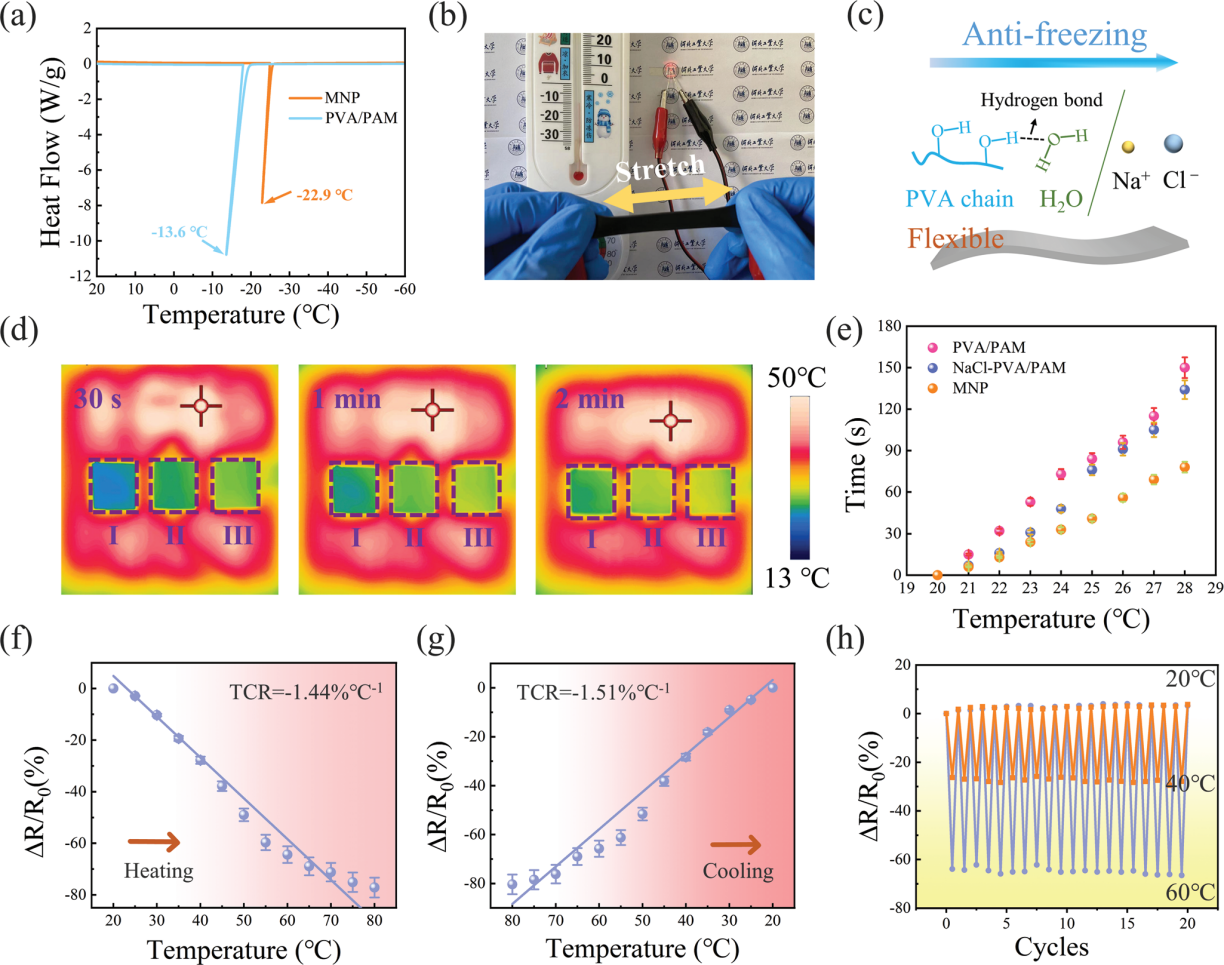
MNP hydrogels were tested for anti‐freezing and thermosensitive properties. a) Differential scanning calorimetry (DSC) of the PVA/PAM and MNP hydrogels from 20 to −60 °C. b) The MNP hydrogel was used as a wire to light up LED lights at sub‐zero temperature. c) Diagram of the anti‐freezing mechanism. d) Infrared (IR) camera images with three different types of hydrogels (I, PVA/PAM; II, NaCl‐PVA/PAM; and III, MNP). e) Temperature versus time plots. Data are presented as mean ± SD (n = 3). f) Relative resistance changes of the MNP hydrogel sensor upon heating and g) cooling from 20 to 80 °C. Data are presented as mean ± SD (n = 3). h) Cyclic variation thermal simulations between 20, 40 and 60 °C.

Soft robots are often exposed to extreme environments, including sub‐zero temperatures, when utilized in real‐world applications. The freezing of free water within the hydrogel disrupts the medium for transport of metal cations, leading to a significant reduction in conductivity. Overcoming the issue of low‐temperature freezing is crucial for the successful application of conductive hydrogels in soft robots. The anti‐freeze property of the conductive hydrogels was analyzed using differential scanning calorimetry (DSC), as shown in **Figure** **4**a. In the temperature range of 20 to −60 °C, the PVA/PAM DN hydrogel exhibited an exothermic peak at −13.6 °C, while the MNP hydrogel showed an exothermic peak at −22.9 °C. The results indicate that the PVA/PAM hydrogel exhibits a certain level of anti‐freezing ability, which is further enhanced by the addition of MWCNT and NaCl in the MNP hydrogel. Figure 4b demonstrates that, when used as a wire with variable resistance, the emitted light from the LED becomes dimmer with increasing tensile strain at low temperatures. Ultimately, the MNP hydrogels demonstrate the ability to withstand temperatures as low as −22 °C without freezing while maintaining their flexibility. To investigate the mechanical properties and ionic conductivity at low temperatures, the MNP hydrogels were placed at −22 °C for 1 h. It observed that the tensile stress was slightly improved at sub‐zero temperatures compared to normal temperatures (Figure S4, Supporting Information). The elongation at break was measured to be 443%, and the breaking strength was found to be 402 kPa. This improvement can be attributed to the strengthening of hydrogen bonds between water molecules and the polymer chains at low temperatures. The conductivity of the hydrogel at −22 °C was measured to be 3.01 S m^−1^ (Figure S5, Supporting Information), with a slight reduction in conductivity due to the low ion transport rate at low temperatures. These results indicate that the MNP hydrogels possess excellent mechanical, electrical properties and strain sensitivity even at low temperatures. These properties arise from the strong hydrogen bonds formed between PVA, PAM and water molecules, inhibiting the tendency of water molecules to freeze. Additionally, the addition of MWCNT and NaCl also lowers the freezing point of water (Figure 4c).

Thermal stimulus perception is the main component to achieve exteroception capability in soft robots. Therefore, the heat transfer properties of the MNP hydrogels were compared with those of the PVA/PAM and NaCl‐PVA/PAM hydrogels. In Figure 4d, the heat transfer properties of the hydrogels were measured using an infrared camera. To assess the amount of heat transfer, the hydrogels of different types were placed on a plate preheated to 37 °C, and their surface temperature was measured. When the MNP hydrogel‐covered plate was exposed to heat, the surface temperature reached 28 °C within 2 min, whereas the other two covered plates failed to reach the target temperature (28 °C) within the same timeframe. The time required to reach 28 °C was measured as follows: the MNP hydrogel took 78 s, the NaCl‐PVA/PAM hydrogel took 134 s, and the PVA/PAM hydrogel took 150 s (Figure 4e). The use of the hybrid conductive filler in the hydrogel significantly improved heat transfer efficiency compared to the other two hydrogels. The significant difference in heat conduction is attributed to the lack of free moving electrons within the polymer, and heat transfer primarily occurs through lattice vibrations. Fortunately, the addition of MWCNT forms an effective thermal conduction network with numerous free electrons, promoting heat transfer and resulting in excellent thermal conductivity of the MNP hydrogel. Therefore, the MNP hydrogels are suitable for sensing thermal stimuli.

The sensitivity of the MNP hydrogel to temperature changes was also investigated. As the temperature increased from 20 to 80 °C, the relative resistance of the hydrogel sensor decreased, indicating a negative temperature coefficient (NTC) behavior of the MNP hydrogel. The temperature coefficient of resistance (TCR) was calculated to be −1.44% °C^−1^ (Figure 4f). This behavior arises from the high aspect ratio and nanometer size of the MWCNT, which allows electrons to move along the axial direction of the MWCNT. Electron transport in MWCNT is influenced by quantum confinement effects. As the temperature increased, the band gap decreased, weakening the confinement effect and increasing electron transport, which in turn led to a decrease in resistance. Figure 4g shows that the ∆ R/R_0_ during cooling exhibited a TCR value of −1.51% °C^−1^, which is approximately a linear relationship. Additionally, cyclic variation thermal simulations were conducted between 20, 40 and 60 °C for 20 experimental cycles (Figure 4h), demonstrating the excellent sensing ability and reproducibility of the MNP hydrogel. This thermal stimulus sensing ability endows the MNP hydrogel with the potential application to assist soft robots in collecting more environmental information.

### 2.5 Deformation and Thermal Stimulus Awareness of MNP‐Integrated Soft Robots

**Figure 5:**
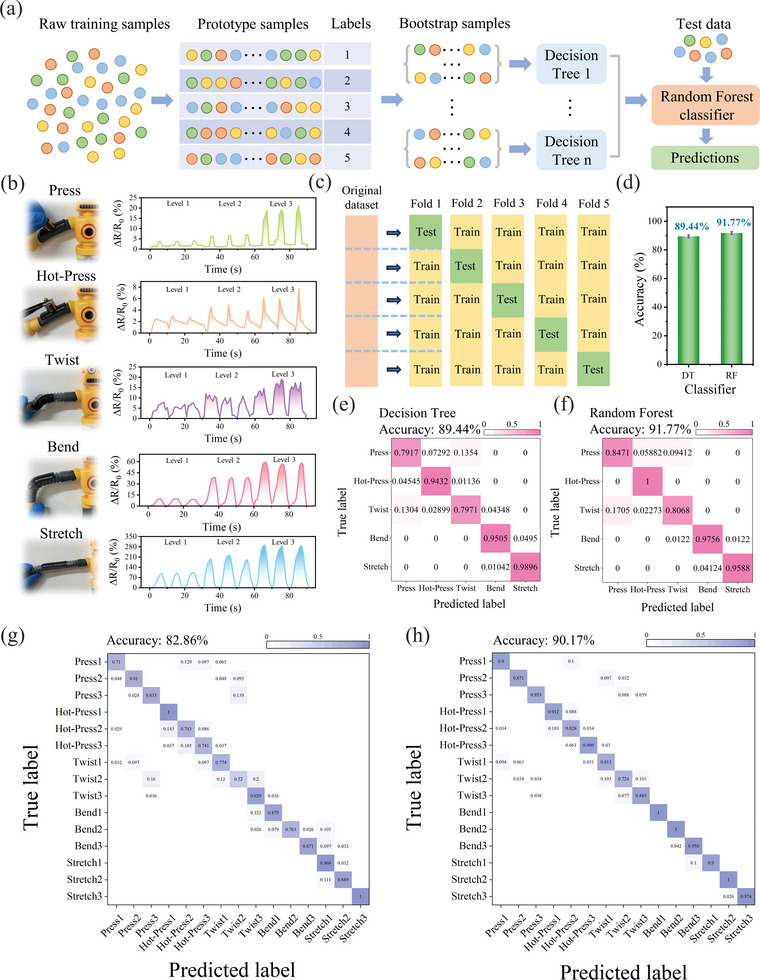
MNP hydrogel‐integrated soft robots for deformation and thermal stimulus awareness. a) Flow diagram illustrating the machine learning process. b) Resistance changes measured during five states (each with three levels). c) Diagram depicting 5‐fold cross‐validation. d) Accuracy comparison of these two algorithms in recognizing five categories of arm states. Data are presented as mean ± SD (n = 3). e) The confusion matrix for decision tree classification. f) The confusion matrix for random forest classification. g) Recognition accuracy for all fifteen conditions combined using the decision tree algorithm, achieving an overall accuracy of 82.86%. h) Recognition accuracy for all fifteen conditions combined using the random forest algorithm, achieving an overall accuracy of 90.17%.

In order to enable intelligent perception, soft robots require automated multimodal identification. Traditionally, the perception of deformation and external thermal stimuli in soft robots usually relies on complex image processing techniques. However, it is now possible to provide multimodal measurements by using MNP hydrogel sensors to realize both exteroception and proprioception and analyze the data in combination with machine learning techniques, which is crucial for soft robots to achieve intelligent perception. ML is a data‐driven approach to empirically approximate the unknown models. It is an appropriate method to bridge the gap between data collected by the MNP sensors and the desired goals. The decision tree algorithm is preferred for its classification capabilities. Meanwhile, multiple decision tree classifiers can be generated to create a random forest, where each decision tree independently makes predictions that are ultimately voted upon to produce the final classification prediction. The processing procedure is outlined in **Figure** **5**a.

To demonstrate the ability of the MNP hydrogel to perceive various deformation states and thermal stimuli, the arm states of the soft robot were classified into five categories: object pressing, hot object pressing, twisting, bending, and stretching. Each category has three loading levels (Figure 5b). These categories were labeled with numbers from one to fifteen to represent the typical response of the sensors under different states. A dataset comprising the five aforementioned states (each with three levels) was compiled, with each signal in Figure 5b repeated 50 times. In order to address the overfitting issue of the model and to improve the generalization ability of the model, we perform data augmentation of the collected data. The data for each level was expanded by flipping and introducing white Gaussian noise for 50 sets respectively, ultimately containing 150 sets of data for each level. The samples of the five states were divided into training and test datasets in a 4:1 ratio. The data from the training samples were used as input for decision tree and random forest classifier to establish the prediction model (Figure 5c). The ability of the algorithm to classify the five states was first verified. In order to achieve optimal selection of the classifier parameters, a grid search method was used for hyper‐parameter optimization. Following fivefold cross‐validation, the optimal decision tree exhibited a maximum depth of 6 and a minimum number of samples for the optimal leaf node of 5 (Figure S6, Supporting Information). The decision tree algorithm achieved an overall recognition accuracy of 89.44% for the five states. Additionally, we established a random forest model to compare classification effects of the two algorithms, employing identical samples and utilizing a grid search approach for hyper‐parameter optimization. The optimal number of trees in the forest was determined to be 50 after fivefold cross‐validation (Figure S7, Supporting Information). Notably, the random forest algorithm outperformed the decision tree algorithm, achieving an overall recognition accuracy of 91.77% (Figure 5d). This is because the random forest algorithm incorporates all the advantages of the decision tree, and the feature importance given by the random forest is more reliable than that given by a single decision tree. In the confusion matrix (Figures 5e,f), it is worth noting that most errors in both algorithms occur when distinguishing between object pressing and twisting. This is attributed to the insignificant curvature difference, and future improvements are expected to modify the pressure‐sensitive surface microstructure.

Furthermore, a test was conducted wherein all five states (each with three levels) were mixed together. The results revealed an overall recognition accuracy of 82.86% for the decision tree algorithm and 90.17% for the random forest algorithm, as shown in Figures 5g,h. It is noteworthy that although the decision tree algorithm demonstrates a certain degree of generalization, its accuracy is not as satisfactory as that of the random forest algorithm. The confusion matrix reveals that the decision tree algorithm still struggles with distinguishing between object pressing and twisting, particularly after each state is divided into three levels, leading to a decrease in recognition accuracy. Conversely, the random forest algorithm maintains a high accuracy during this period. Hyper‐parameter optimization using the grid search method confirms that the optimal number of trees in the forest at 75 after fivefold cross‐validation (Figure S8, Supporting Information). Overall, our study demonstrates that the integration of ML‐assisted methods has enabled soft robots to perceive deformation and thermal stimuli.

## 3 Conclusion

We designed and fabricated a novel flexible MNP hydrogel sensor and have successfully integrated it into a soft robot to achieve exteroception and proprioception. It is based on hybrid electronic/ionic conductivity, achieving high sensitivity and a wide strain range through the homogeneous distribution of MWCNT and NaCl in the PVA/PAM DN hydrogel. Additionally, the MNP hydrogel maintains exceptional mechanical and electrical properties at sub‐zero temperatures, and demonstrates excellent thermal stimulus perception capabilities. Importantly, we have leveraged ML technology to analyze MNP‐generated data, enabling the identification of different states such as object pressing, hot object pressing, twisting, bending and stretching. This infusion of ML techniques endows soft robots with awareness and basic intelligence capabilities. The MNP hydrogel and its integration with ML techniques lay a strong foundation for soft robots to effectively perceive their own environmental states. We believe that this well‐designed MNP hydrogel holds great potential for real‐world applications in soft robots.

## 4 Experimental Section

### Materials

Unless otherwise indicated, the primary components utilized in this study include MWCNT, NaCl and PVA/PAM hydrogel. Among them, MWCNT, purchased from Taixi Technology Co., Ltd, was used as electronic conductors, which have an average diameter of 60–150 nm and a length of 9–15 µm (Figures S1 and S2, Supporting Information). Sodium dodecyl sulfate (SDS; Aladdin, AR) was used as dispersant. NaCl (Sinopharm, GR) was employed as the ionic charge carrier. Polyvinyl alcohol (PVA; Aladdin, 1799, alcoholysis degree: 98–99%) and acrylamide (AM; Aladdin, AR) were the base materials of the hydrogel. N,N′‐methylenebis acrylamide (MBAA; Aladdin,99%) was used as the cross‐linking agent for the AM gel. Ammonium persulfate (APS; Aladdin, 98%) and N,N,N′,N′‐tetramethyl ethylenediamine (TEMED; Aladdin, 99%) were the thermal initiator and accelerator for the gelation reactions, respectively. Deionized water (18.2 mΩ at 25 °C), copper (Cu) electrodes and wires were also used in the experiments.

### Synthesis of MNP, PVA, and PAM Hydrogels

The MNP hydrogel was synthesized by dissolving MWCNT/NaCl and PVA/PAM in deionized water. MWCNT and SDS were mixed at a mass ratio of 1:1, and the MWCNT dispersion was obtained through mechanical stirring and ultrasonic dispersion. Subsequently, the MWCNT mixture was blended with PVA and magnetically stirred at 80 °C for 1 h until the PVA was completely dissolved (Solution 1, 40 mL). At room temperature, NaCl was mixed with the AM monomer for 1 h under magnetic stirring (Solution 2, 40 mL). Then, the cross‐linker MBAA (0.06 wt% with respect to the AM monomer) and the initiator APS (0.16 wt% with respect to the AM monomer) were added to Solution 2. Solutions 1 and 2 were mixed and stirred at room temperature for 1 h. After defoaming, TEMED (0.25 wt% with respect to the weight of the AM monomer) was added as the accelerator. The mixed liquid was poured into a specific mold at 60 °C for 2 h to accelerate PAM cross‐linking. Subsequently, the mold went through 4 h of freezing at −26 °C followed by 4 h of warming up to 26 °C. The MNP hydrogel was finally obtained after three cycles of freezing and thawing. The MWCNT/NaCl‐PVA hydrogel was obtained by adding NaCl to solution 1 after three cycles of freezing and thawing. The MWCNT/NaCl‐PAM hydrogel was obtained by adding the MWCNT dispersion and TEMED to solution2 and holding at 60 °C for 2 h.

### Characterization of MWCNT and MNP Hydrogel

The morphology of the ultrasonically dispersed MWCNT was observed using a field‐emission scanning electron microscope (SEM, FEI‐NOVA NANOSEM 230). The purity of MWCNT was analyzed by Raman spectroscopy (Renishaw‐invia RM‐1000). The MNP hydrogel was freeze‐dried in a low temperature test chamber to remove water completely and then observed with SEM. The functional groups in the MNP hydrogel were identified by Fourier transform infrared spectrometry (FTIR, Thermo Scientific Nicolet IS5) within the 400–4000 cm^−1^ range. The crystalline properties of the MNP hydrogel were characterized using the X‐ray diffractometer (XRD, Rigaku Smart Lab 9 kW) at room temperature. The freezing point of the MNP hydrogel was determined using differential scanning calorimetry. The sample temperature was cooled from 20 to −60 °C at a rate of −2 °C min^−1^.

### Mechanical and Electrical Tests

Mechanical testing was carried out using a universal testing machine (XJ810, Xiangjie) equipped with a 1000 N load cell. The stretched samples have dimensions of 50 mm × 10 mm ×1 mm (length, width and thickness, respectively). A gauge length of 30 mm was used for strain measurement, and the loading and unloading rates were set at 20 mm min^−1^. Tensile stress (*σ*) was calculated by dividing the force (*F*) by the cross‐sectional area (*A*) using the formula σ  =  *F*/*A*. Strain (*ε*) was calculated by dividing the length change (*∆L*) by the original length (*L*_0_): expressed as ε  =  Δ*L*/*L*_0_.

The resistance of the MNP hydrogel was measured by a digital source meter (Victor, 8165A). The ionic conductivity of the MNP hydrogel was determined using alternating current impedance (AC impedance) on an electrochemical workstation (Autolab Electrochemical Workstation‐PGSTAT302N, Metrohm). The frequency range was set from 0.01 Hz to 1 MHz, with a voltage amplitude of 5 mV.

### Sensing Tests

During the uniaxial tension tests, the resistance of MNP hydrogels (*R*) was simultaneously measured using a digital source meter (Victor, 8165A). Gauge factor (GF) that characterizes the tensile strain sensitivity of the hydrogel‐based sensors was obtained as the ratio of the relative resistance change rate to the applied strain: *GF*  = [(*R*‐*R*_0_)/*R*_0_] /ε, where *R_0_* is the initial resistance.

To compare the heat transfer rate of different hydrogels, namely PVA/PAM hydrogel, NaCl‐PVA/PAM hydrogel, and MNP hydrogel, the samples were placed on a plate preheated at 37 °C, and their surface temperature was measured using an infrared (IR) camera. Simultaneously, the temperature coefficient of resistance (TCR) was defined as: *TCR*  = (*R*‐*R*_0_)  / *R*_0_ / (*T*_2_‐*T*_1_), where *R*_0_ is the resistance at the initial temperature *T*_1_, and *R* is the resistance at the test temperature *T*_2_.

### Data Measurement and Training

The hydrogel sensor exhibited a change in resistance when subjected to mechanical deformation and thermal stimuli, which was measured by a digital source meter. The sensor was connected to the soft robot via the medical polyurethane (PU) tape, with copper serving as the electrode. From a long‐term perspective, employing silver electrodes was recommended, as prolonged exposure of copper surfaces to water can lead to chemical reactions that may affect the signal. The states of the soft robot were classified into five categories: object pressing, hot object pressing, twisting, bending, and stretching, with each category comprising three loading levels. After data acquisition, the data points were segmented based on the type of event with the same duration. The model was built using the decision tree and the random forest algorithms.

### Statistical Analysis

All raw statistical data were collected by digital source meter (Victor, 8165A). During the feature extraction process, the mean, standard deviation, median, and maximum values were obtained by imputing the original data into Origin 2019b and utilizing its statistical module to generate the respective statistical data. All results have been presented as mean with standard deviation (mean ± SD). The sample size (n) of independent repeated experiments for each statistical analysis was given in the figure legends. Furthermore, the data used for machine learning purposes was segmented into data points based on event types of the same length of time, with each event repeated 50 times. Python code, implemented on the PyCharm Community Edition 2022.1 platform, was utilized to augment the data by flipping data points and introducing white Gaussian noise, resulting in 150 sets of data per event.

## Conflict of Interest

The authors declare no conflict of interest.

## Supporting information

Supporting Information

## Data Availability

The data that support the findings of this study are available from the corresponding author upon reasonable request.
