# Supplementary material for: Machine Learning Assisted Electronic/Ionic Skin Recognition of Thermal Stimuli and Mechanical Deformation for Soft Robots
Source: Adv Sci (Weinh). 2024 Jun 12;11(30):2401123. doi: 10.1002/advs.202401123 (PMC11321626; doi:10.1002/advs.202401123)
Supplement: Supplementary file 1 — Supporting Information [file ADVS-11-2401123-s001.docx]

Supporting Information

**Machine Learning Assisted Electronic/Ionic Skin Recognition of Thermal Stimuli and Mechanical Deformation for Soft Robots**

*Xuewei Shi^1^, Alamusi Lee^1^*, Bo Yang^1^*, Huiming Ning^2^, Haowen Liu^1^, Kexu An^1^, Hansheng Liao^1^, Kaiyan Huang^3^, Jie Wen^1^, Xiaolin Luo^4^, Lidan Zhang^5^, Bin Gu^3^ and Ning Hu^1,6,7^**

X.W. Shi^1^, A.L.M.S. Lee^1^*, B. Yang^1^*, H.W. Liu^1^, K.X. An^1^, H.S. Liao^1^, J. Wen^1^, N. Hu^1,6,7^*

^1^School of Mechanical Engineering, Hebei University of Technology, Tianjin, 300401, China.

^6^State Key Laboratory of Reliability and Intelligence Electrical Equipment, Hebei University of Technology, Tianjin, 300130, China.

^7^Key Laboratory of Advanced Intelligent Protective Equipment Technology, Ministry of Education, Hebei University of Technology, Tianjin, 300401, China.

H.M. Ning^2^

^2^College of Aerospace Engineering, Chongqing University, Chongqing 400044, China.

K.Y. Huang^3^, B. Gu^3^

^3^School of Manufacturing Science and Engineering, Southwest University of Science and Technology, 59 Qinglong Road, Mianyang 621010, China.

X.L. Luo^4^

^4^First Teaching Hospital of Tianjin University of Traditional Chinese Medicine, National Clinical Research Center for Chinese Medicine Acupuncture and Moxibustion, Tianjin, 300381, China.

L.D. Zhang^5^

^5^School of Basic Medicine, Chongqing Medical University, Chongqing, 400042, China.

E-mail: alamusi@hebut.edu.cn; boyang@hebut.edu.cn; ninghu@hebut.edu.cn.

Keywords: Electronic/ionic conductive hydrogel, mechanical deformation, thermal stimuli, machine learning

**Figure S1.** SEM images of MWCNT. The average diameter is 60-150 nm and the average length is 9-15 μm.

Raman spectroscopy is a powerful tool for characterizing the degree of graphitization or structural orderliness of carbon nanotubes. As shown in Figure S2, Raman spectroscopy was implemented to assess the MWCNT utilized in this study. Two Raman peaks of D band and G band could be obtained as 1363 cm^-1^ and 1590 cm^-1^. The peak of D band reflects the disordered graphite structure and the peak of G band is used to illustrate the degree of graphitization of the MWCNT. The ratio I_D_/I_G_ of the two peak intensities can be used to indicate the extent of defects. A higher ratio indicates more defects and more disorganized structure of the MWCNT. When compared to other studies ^[1, 2]^, the I_D_/I_G_ in this study is 0.19, which is a relatively low value. This finding points to the fact that the MWCNT are structurally well-ordered and possess few defects.

**Figure S2.** Raman spectrum of MWCNT.


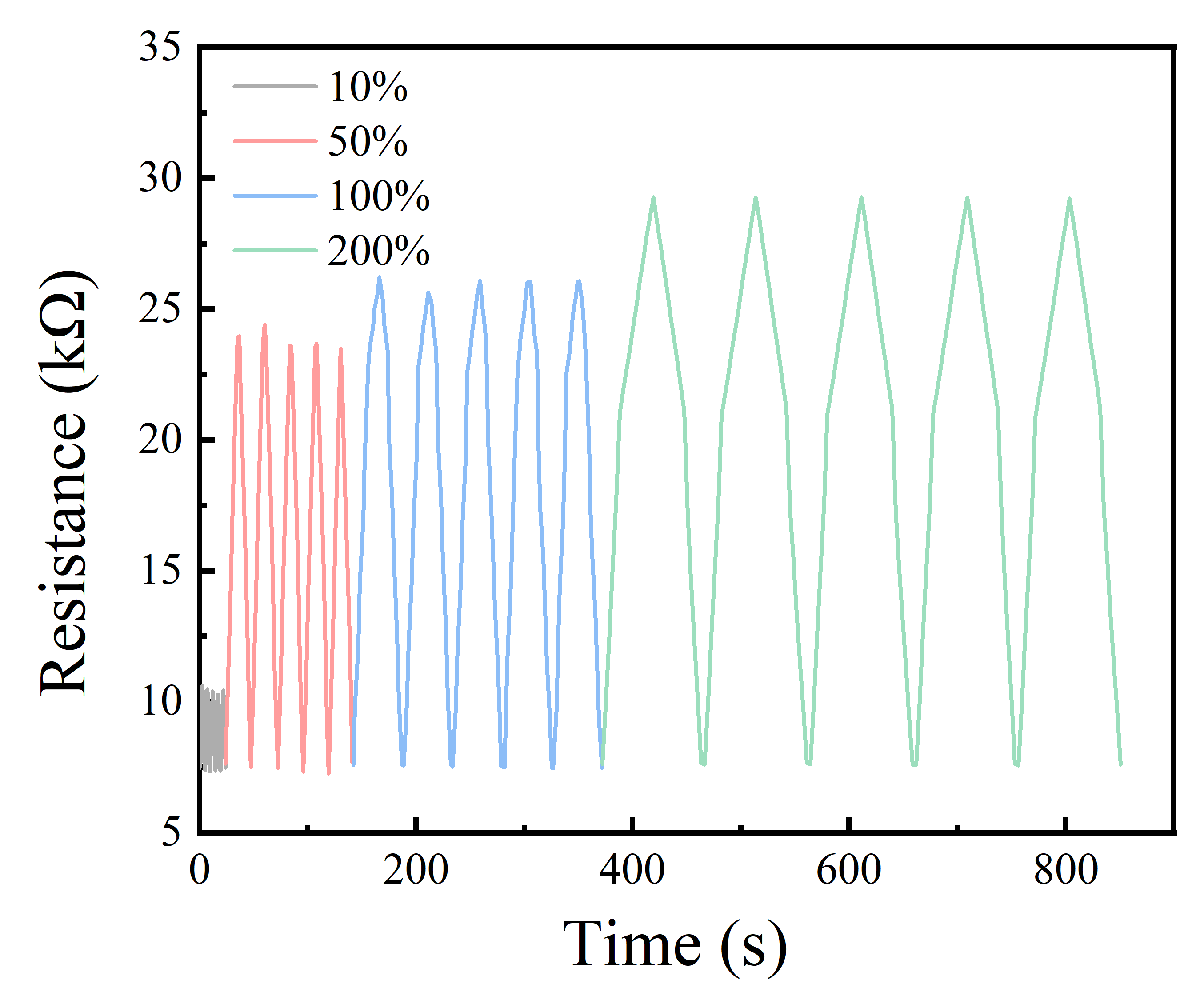


**Figure S3.** Resistance changes under various tensile strains (10%, 50%, 100%, and 200%).

**Figure S4.** The tensile stress-strain curve of MNP hydrogel at sub-zero temperature state (elongation at break is 443% and tensile strength is 402 kPa).


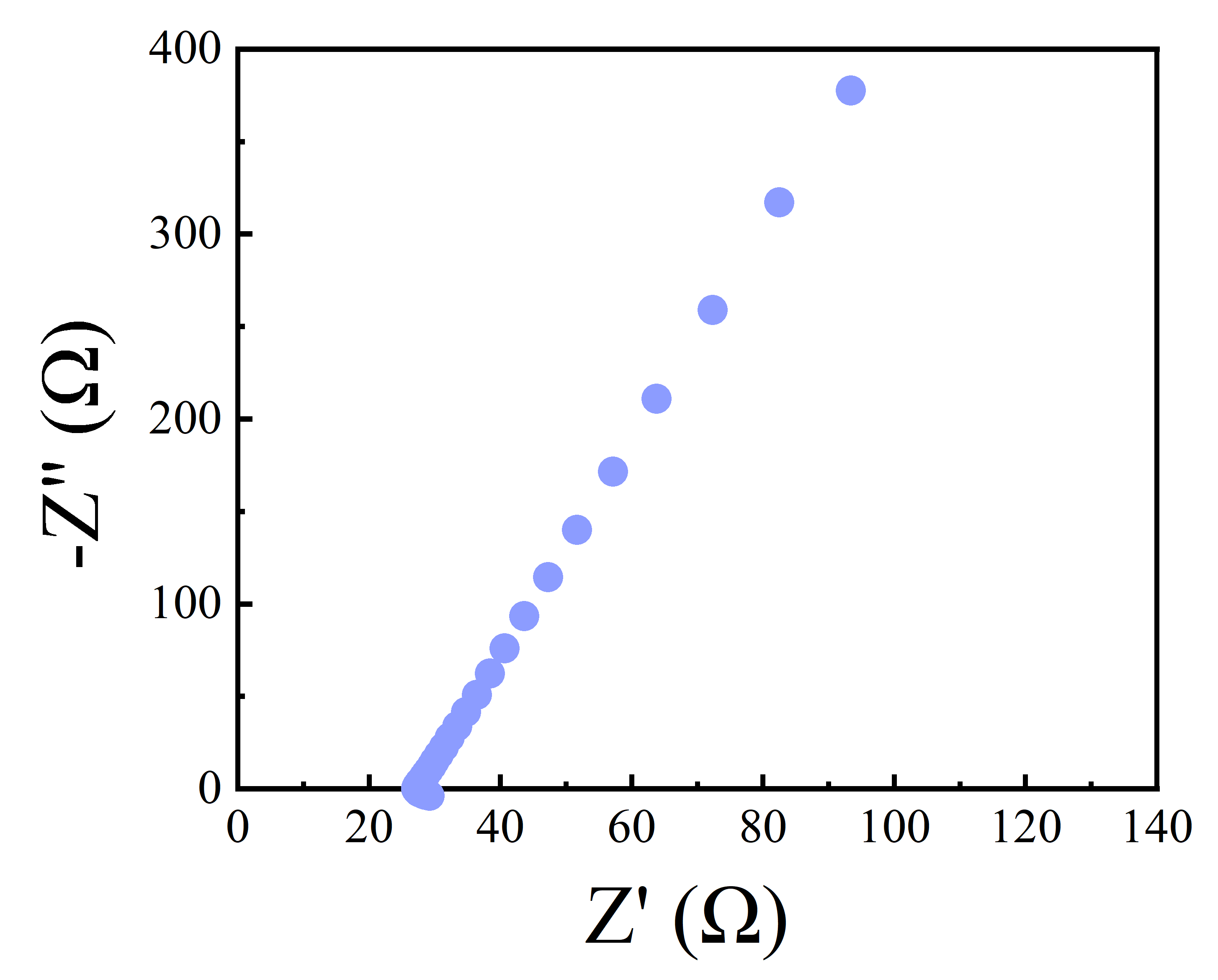


**Figure S5.** Electrochemical impedance spectra of MNP hydrogels at sub-zero temperature state.


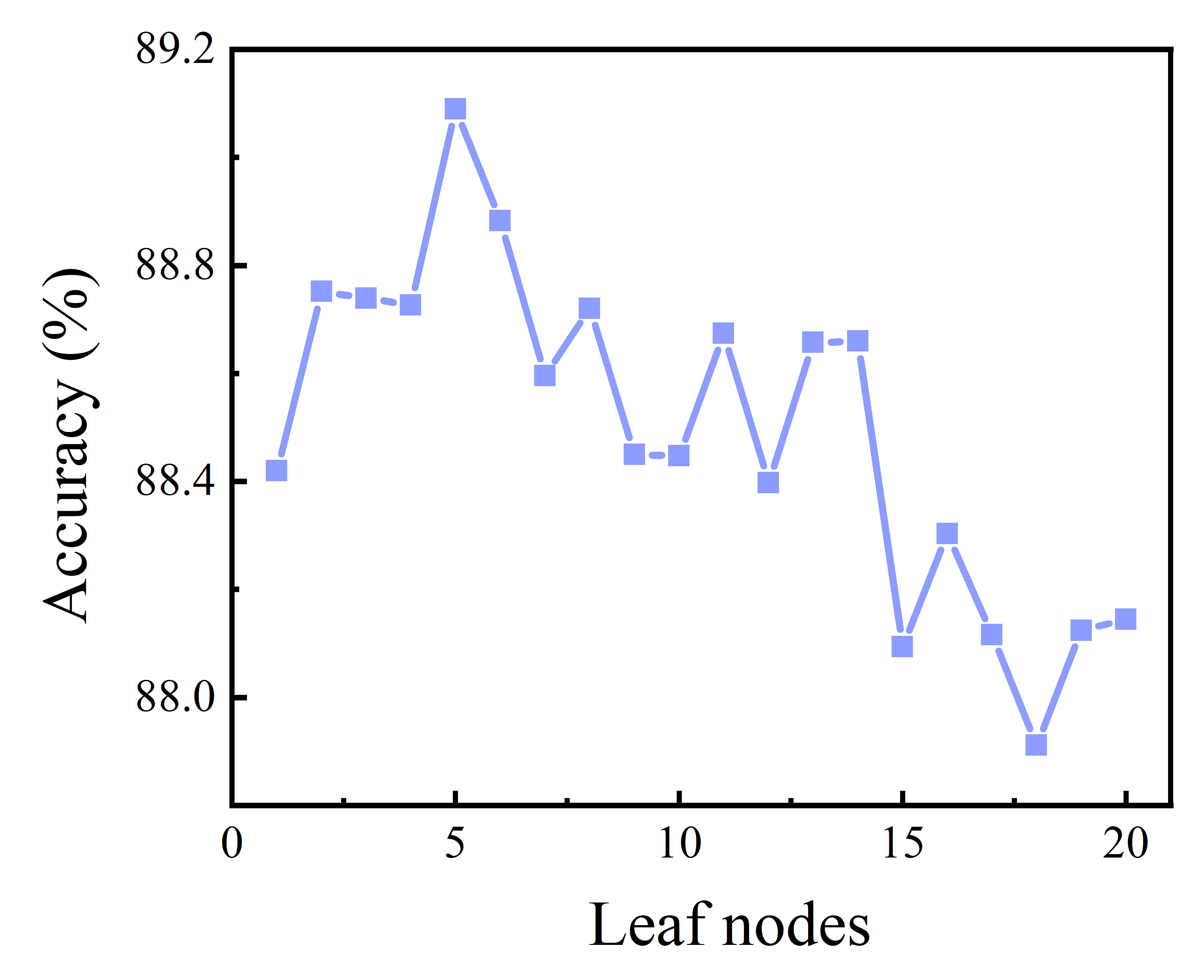


**Figure S6.** Accuracy of the decision tree algorithm corresponding to different number of leaf nodes.


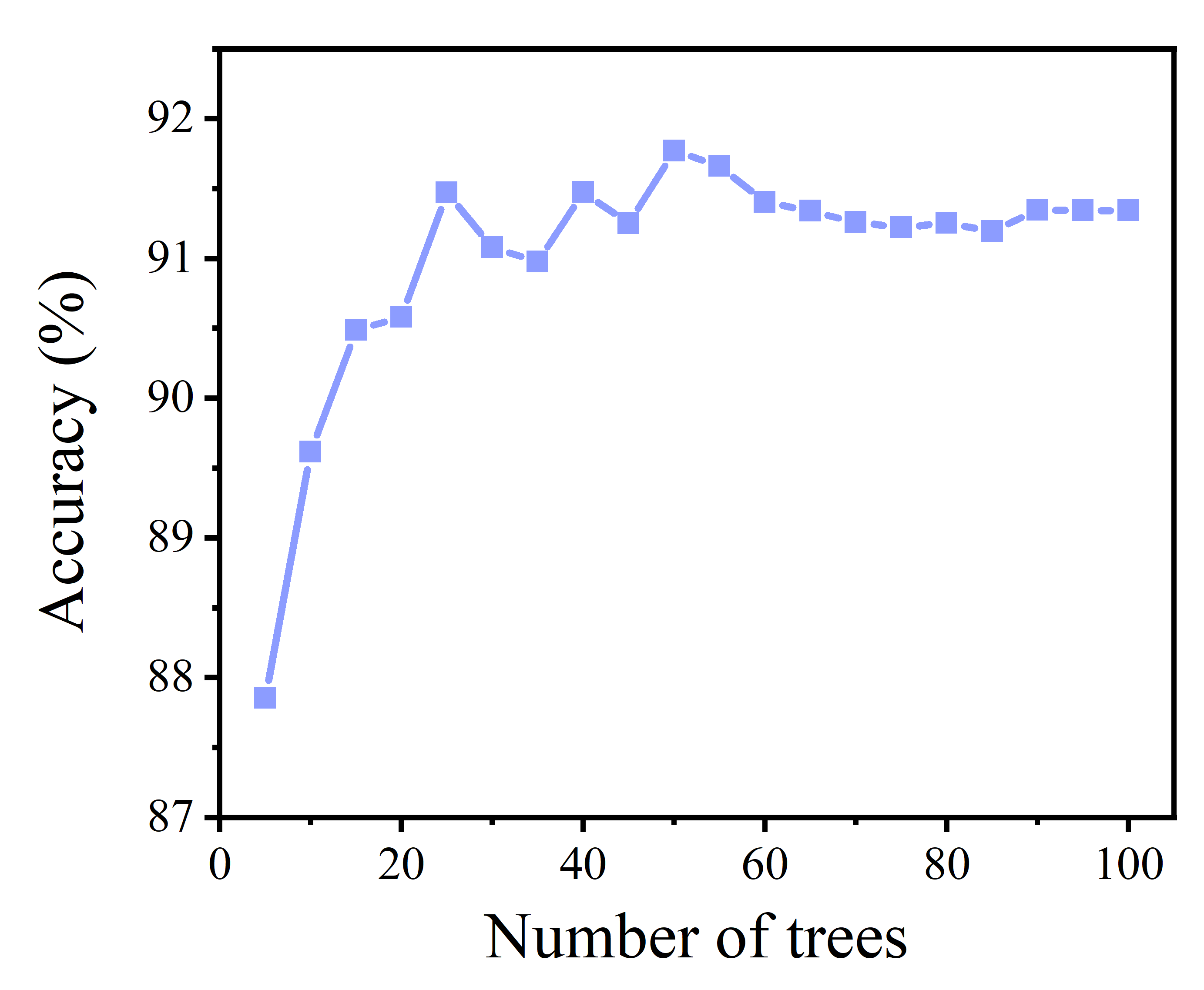


**Figure S7.** Accuracy of the random forest algorithm corresponding to different number of trees.


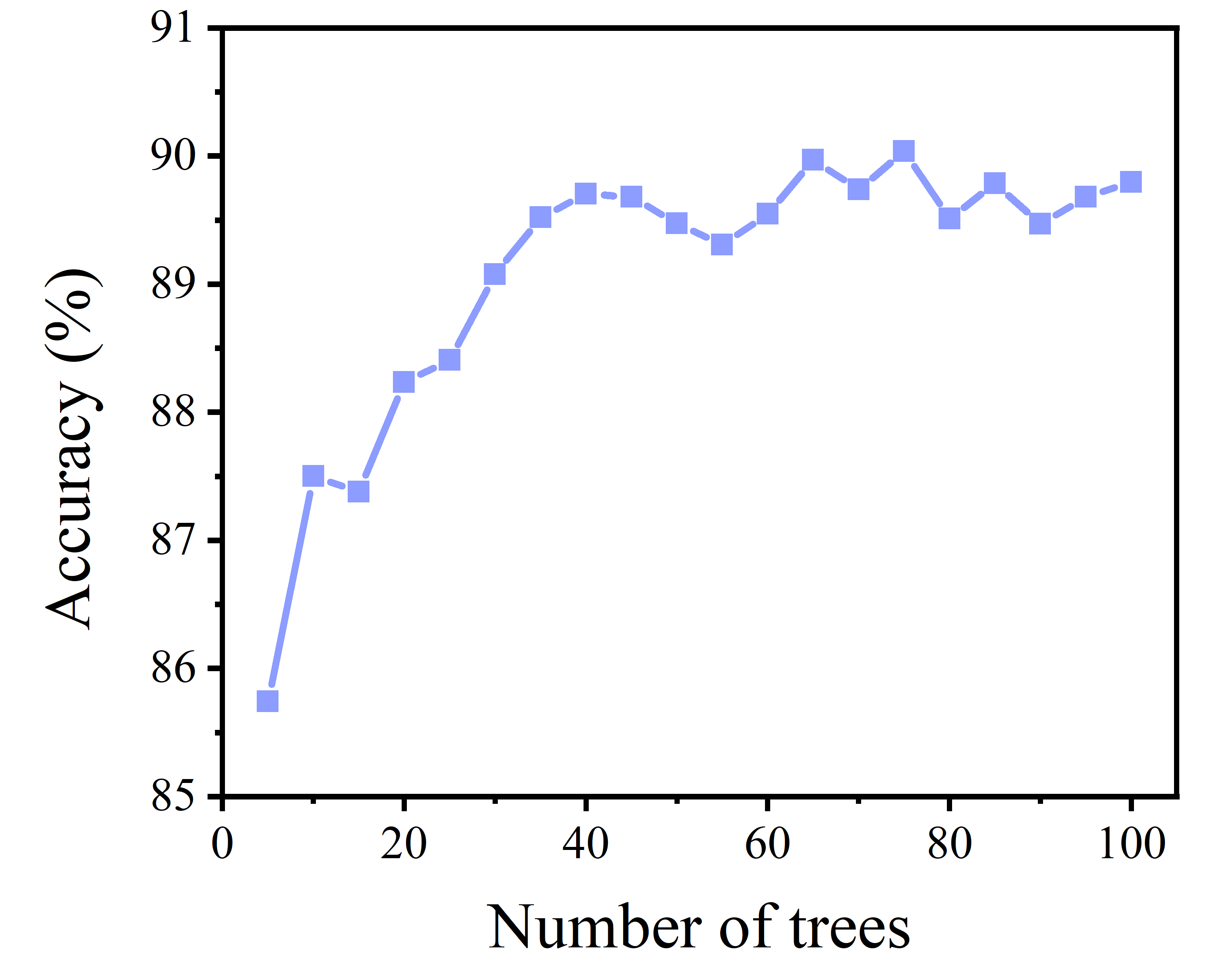


**Figure S8.** Accuracy of the random forest algorithm corresponding to different numbers of trees for categorizing five states (three levels each).

**Table S1.** Comparison of mainstream electronic conductive hydrogel strain sensors.

| Materials | Methods | Strain Range (%) | Gauge Factor (GF) | Linear Relationship | Reference |
| --- | --- | --- | --- | --- | --- |
| NCB/fluorocarbon elastomer | Compression-molding | 10 | 11.2 | yes | 37 |
| rGO/CNT/PU@EP | Layer-by-layer assembly | 0.8 | 5.2 | yes | 38 |
| GNP/PDMS | Self-sacrificing templating | 70 | 5.49 | yes | 39 |
| CNF/GNP/PDMS | Spin coating | 50 | 3.5 | yes | 40 |
| CBNP/styrene-butadiene rubber | Cold emulsion polymerization | 15 | 3 | yes | 41 |
| MWCNT/epoxy resins | Casting fifilm | 0.2 | 2.61 | yes | 42 |
| MWCNT/PDMS | Coating | 80 | 2.26 | yes | 43 |
| Ppy/GNP/CB/SR | Solution casting | 100 | 1.4 | no | 44 |

**Table S2.** Comparison of mainstream ionic conductive hydrogel strain sensors.

| Materials | Methods | Strain Range (%) | Gauge Factor (GF) | Linear Relationship | Reference |
| --- | --- | --- | --- | --- | --- |
| HEC/PAM/PAMPS/LiCl | Thermal initiation | 50 | 0.74 | yes | 45 |
|  |  | 200 | 2.28 |  |  |
| PVA-SbQ/SA/FeCl3/Gly | UV irradiation and immersion | 100 | 0.61 | yes | 46 |
|  |  | 500 | 2.49 |  |  |
| PAAm/PDMS-LiCl | 3D printing extrusion | 50 | 0.84 | yes | 47 |
| LiCl/PVA/CS/PAAm | Thermal polymerization and F-T cycle | 120 | 0.93 | yes | 48 |
| Cellulose/NaCl | Solution casting | 150 | 0.95 | yes | 49 |
| PAA/PVA-FeCl3 | Free radical polymerization and F-T cycle | 150 | 0.12 | yes | 50 |
| Cassava starch/rubber/LiCl/glycerol | Thermal polymerization | 400 | 0.906 | yes | 51 |

**Reference**

[1] K. Behler, S. Osswald, H. Ye, S. Dimovski, Y. Gogotsi, *J*. *Nanopart*. *Res*. **2006**, *8*, *615*.

[2] J. Zhao, Y. Z. Zhang, Y. J. Su, X. L. Huang, L. M. Wei, E. S. W. Kong, Y. F. Zhang, *Diamond Relat*. *Mater*. **2012**, *25*, *24*.
